# Reply to Correspondence: Response to a case report: idiopathic hypereosinophilic syndrome in remission with benralizumab treatment after relapse with mepolizumab

**DOI:** 10.1002/rcr2.708

**Published:** 2021-01-07

**Authors:** Koki Fujii, Hidenori Takahashi, Nami Hayakawa, Yoshinobu Iwasaki

**Affiliations:** ^1^ Division of Respiratory Medicine, Department of Medicine Showa General Hospital Tokyo Japan; ^2^ Department of Plastic Surgery Tokyo Women's Medical University Hospital Tokyo Japan

## Abstract

See related Letter

## Reply

*From the Authors:*

We are grateful for the correspondence by Requena et al. [1]. We explain some discrepancies pointed out in the correspondence.

First, the dose of mepolizumab for this patient was 100 mg subcutaneously once every four weeks. The dose of benralizumab was 30 mg SC once every four weeks for the first three doses followed by once every eight weeks. As they indicated, the dose of mepolizumab for this patient was lower than the dose in the phase III study about the therapeutic effect of mepolizumab on hypereosinophilic syndrome (HES). The treatment effect of mepolizumab was potentially underestimated. Furthermore, due to the limitation of the number of words, the medical history was summarized, and details such as the timing of steroid tapering were omitted.

The data from Flood‐Page et al. we quoted are for mild asthma, and 750 mg of mepolizumab is different from the dose used for this patient [2]. Therefore, it cannot be generally applied to this HES patient, but it is considered to be important data when examining the therapeutic effect of mepolizumab.

The course of treatment was shown in figure 2 in our case report. It contained only data from the first visit to 15 months later, but the patient is still in remission at the time of writing (December 2020). Mepolizumab had a recurrence after about six months of use, whereas benralizumab maintains remission for about 26 months.

We are sorry that there is a correction in the text. We correct the sentence “benralizumab produces a median decrease in the content of peripheral blood eosinophils from a baseline of 95.8% in the airway mucosa, 89.9% in sputum, and 100% in the bone marrow” to the sentence “benralizumab produces a median reduction of 95.8% in airway mucosal eosinophil, 89.9% in sputum, and 100% in blood” [3]. No lung biopsy or bronchoalveolar lavage was performed due to poor respiratory condition. If it could be performed, more meaningful information would have been obtained.

As Requena et al. stated, it is premature to formulate any conclusion on whether mepolizumab or benralizumab is more effective in HES based on a single case report. It is desirable that more observations and data could be gathered. We believe this case report, along with more reliable data from other patients with idiopathic‐HES (I‐HES), may help in the development of more efficacious therapeutic interventions for I‐HES in the future.
